# Anti-inflammation effects of picroside 2 in cerebral ischemic injury rats

**DOI:** 10.1186/1744-9081-6-43

**Published:** 2010-07-09

**Authors:** Yunliang Guo, Xinying Xu, Qin Li, Zhen Li, Fang Du

**Affiliations:** 1 Institute of Cerebrovascular Diseases, Affiliated Hospital of Qingdao University Medical College, Qingdao 266003, China; 2 Department of Emergency, Yantai Yuhuangding Hospital, Yantai 264000, China; 3 Department of Neurology, Anhui Municipal Hospital, Hefei 230011, China

## Abstract

**Background:**

Excitatory amino acid toxicity, oxidative stress, intracellular calcium overload, as well as inflammation and apoptosis are involved in the pathological process after cerebral ischemic reperfusion injury. Picrodide 2 could inhibit neuronal apoptosis and play anti-oxidant and anti-inflammation role in cerebral ischemia/reperfusion injuries, but the exact mechanism is not very clear. This study aims to explore the anti-inflammation mechanism of picroside 2 in cerebral ischemic reperfusion injury in rats.

**Methods:**

The middle cerebral artery occlusion reperfusion models were established with intraluminal thread methods in 90 adult healthy female Wistar rats. Picroside 2 and salvianic acid A sodium were respectively injected from tail vein at the dosage of 10 mg/kg for treatment. The neurobehavioral function was evaluated with Bederson's test and the cerebral infarction volume was observed with tetrazolium chloride (TTC) staining. The apoptotic cells were counted by in situ terminal deoxynucleotidyl transferase-mediated biotinylated deoxyuridine triphosphate nick end labeling (TUNEL) assay. The immunohistochemistry stain was used to determine the expressions of toll-like receptor 4 (TLR4), nuclear transcription factor κB (NFκB) and tumor necrosis factor α (TNFα). The concentrations of TLR4, NFκB and TNFα in brain tissue were determined by enzyme linked immunosorbent assay (ELISA).

**Results:**

After cerebral ischemic reperfusion, the rats showed neurobehavioral function deficit and cerebral infarction in the ischemic hemisphere. The number of apoptotic cells, the expressions and the concentrations in brain tissue of TLR4, NFκB and TNFα in ischemia control group increased significantly than those in the sham operative group (*P *< 0.01). Compared with the ischemia control group, the neurobehavioral scores, the infarction volumes, the apoptotic cells, the expressions and concentrations in brain tissue of TLR4, NFκB and TNFα were obviously decreased both in the picroside 2 and salvianic acid A sodium groups (*P *< 0.01). There was no statistical difference between the two treatment groups in above indexes (*P *> 0.05).

**Conclusions:**

Picroside 2 could down-regulate the expressions of TLR4, NFκB and TNFα to inhibit apoptosis and inflammation induced by cerebral ischemic reperfusion injury and improve the neurobehavioral function of rats.

## Background

Excitatory amino acid toxicity, oxidative stress, intracellular calcium overload, as well as inflammation and apoptosis, are involved in the pathological process after cerebral ischemic reperfusion injury[1]. Among these, the inflammatory cytokines activate nuclear transcription factor κB (NFκB) through toll-like receptor 4 (TLR4)-NFκB signal transduction pathways, promote target gene activation, such as tumor necrosis factor α (TNFα), interleukin (IL), intercellular adhesion molecule (ICAM), and ultimately induce neuronal apoptosis[2,3]. *Picrorhiza scrophulariflora *belongs to the plant family composed of picroside 1, 2 and 3, of which picroside 2 is one of the most effective components extracted from the dried rhizome and roots of *Picrorhiza kurrooa *Royle ex Benth[4] and *Picrorhiza scrophulariae flora *Pennell [5]. It has been traditionally used to treat disorders of the liver, upper respiratory tract diseases, dyspepsia, chronic diarrhea and scorpion sting[5,6]. Current researches on picroside 2 are focused on its neuroprotective[7], anti-apoptotic, anti-cholestatic, anti-oxidant, anti-inflammation, immunemodulating activities[8,9]. It has been confirmed that picroside 2 could enhance nerve growth factor-induced PC12 cell axon growth, reduce H_2_O_2 _induced PC12 cell damage and improve cell survival *in vitro *[10-13]. Animal experiments showed that the extractive of *Picrorhizae *could inhibit cell apoptosis in ischemic penumbra and improve neurobehavioral function in middle cerebral artery occlusion and reperfusion (MCAO/R) rats[14,15]. Our previous experiments[16-19] indicated that picroside 2 could inhibit the expressions of inducible nitric oxide synthase (iNOS), nuclear transcription factor κB (NFκB) and inhibitor of NFκB (IκB), and reduce the expressions of Caspase-3 and poly ADP-ribose polymerase (PARP) to inhibit the neuronal apoptosis and thus improve the neurobehavioral function of rats with cerebral ischemia reperfusion injury. However, it is poorly understood that how many ingredients there are in the extract *Picrorhizae *and which one can play physiological role. In the study, we established the experimental MCAO/R model to investigate the effects of picroside 2 via tail vein injection on TLR4-NFκB signal transduction pathway and cell apoptosis, and to explore its anti-inflammatory mechanism in cerebral ischemic reperfusion injury.

## Methods

### Establishment of animal models

The total of 90 adult female Wistar rats, weight 230 - 250 g, SPF grade, were granted by Qingdao Laboratory Animal Center (SCXK (LU) 20030010). The guidance suggestions for care of laboratory animals was followed according to the *Guidelines for caring for experimental animals *published by the Ministry of Science and Technology of the People's Republic of China. All animals were reared in the laboratory environment, allowed free access to food and water in room temperature and humidity-controlled housing with natural illumination for a week, and fasted 12 h before operation. Fifteen rats were randomly selected as a sham operated group (SO), and the rest 75 rats were subjected to the experimental middle cerebral artery occlusion 2 h and reperfusion 22 h (MCAO/R) models with intraluminal monofilament suture from left external-internal carotid artery [20,21]. At 2 h after the operation, those with left Horner sign, right anterior limb flexible or circling towards right, were considered as symbols of successful models. Core body temperature was monitored with a rectal probe and maintained at 36°C-37°C using a homeothermic blanket control unit (Qingdao Apparatus, China) during and after the surgery operation. As a result, 45 successful models, as the candidate, were randomly divided into a ischemia control group (IC, n = 15), a salvianic acid A sodium group (SA, n = 15) and a picroside treated group (PT, n = 15) according to drug administration. Rats in the SO group were experimented with the same surgical procedure but the monofilament was advanced only about 10 mm and immediately withdrew. The 30 dead or unsuccessfully occluded rats were excluded from this experiment.

### Intervention study

**Figure 1:**
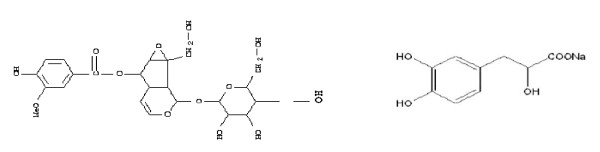
**The chemical structure formula of picroside 2 (left) and salvianic acid A sodium (right)**.

According to *the Pharmacopoeia of the People's Republic of China*, the molecular formula of picroside 2 is C_23_H_28_O_13_, molecular weight is 512.48; the molecular formula of salvianic acid A sodium is C_9_H_9_O_5_Na, molecular weight is 221.17. The chemical structure formulae[22] are shown as figure 1:

Picroside 2 (Tianjin Kuiqing Medical Technology Co. Ltd., CAS No: 39012-20-9, purity > 98%), as a treatment drug, was diluted into 1% injection with 0.1 mol/L PBS, and salvianic acid A sodium (Shanghai Huyun Medical Technology Co. Ltd., CAS No: 23028-17-3, purity > 98%) was also diluted into 1% solution as a positive control drug. According to Xiao's report [23], the rats in PT group were administrated picroside 2 (10 mg/kg) 250 μl via tail vein at ischemia 2 h prior to reperfusion with a micro-syringe. Rats in SA group were given salvianic acid A sodium (10 mg/kg) 250 μl, while those in IC group and SO group were simultaneously suffered 0.1 mol/L PBS 250 μl.

### Neurobehavioral function assessment

All animal neurobehavioral tests were performed at ischemia 2 h and reperfusion 22 h by an investigator who was blinded to the experiment according to the standard of Bederson's report[24]. 0 score: no neurological functional impairment; 1 score: any part of forepaw flexed (positive for tail suspension test) without other abnormal signs; 2 scores: lateral pushing resistance ability decreased (positive for lateral pushing experiment), accompanied with forepaw flexion without circling tendency; 3 scores: same behavior as those for 2 scores, in addition to spontaneous rotation (circling around paralyzed limbs during free activity). The higher the score is gotten, the worse the neurobehavioral dysfunction is appeared, vice versa.

### TTC staining

To determine the infarction volume, five rats in each group were deeply anesthetized by 10% chloral hydrate (300 mg/kg) and decapitated at given time after MCAO/R. The brain tissue was removed and successivelly sliced into 2.0 mm-thick coronal sections. The total of five brain slices were incubated in 2% TTC solution for 10 min at 37°C and then transferred into 4% formaldehyde solution for fixation. The normal brain tissue appeared uniform red while the infarction region showed white. The infarction volumes were calculated in a blinded manner with Adobe PhotoShop CS analysis system. The data were expressed as the percentage of the infarction volume/the ipsilateral hemisphere volume (%) at the coronal section of optic chiasma.

### Neuronal apoptosis assay

Five rats in each group were deeply anesthetized by 10% chloral hydrate (300 mg/kg), reperfused with sodium chloride and 4% formaldehyde 200 ml from the heart into the aorta, and then decapitated at given time. Brain samples were chosen from frontal fontanelle 2 mm to occipital fontanelle 4 mm by a stereotaxic point, post-fixed in 4% formaldehyde for 2 h, dehydrated in alcohol gradually, hyalinized by dimethylbenzene, embedded in paraffin, then sectioned at a thickness of 5 μm, adhered to the sections prepared with poly-L-Lysine, and finally stored at 4°C. To detect cell apoptosis, TUNEL staining was performed according to the protocol of DendEnd Fluorometric TUNEL Detection System (Santa Cruz Co. Ltd.). Coronal paraffin sections described as above were deparaffinaged by dimethylbenzene, hydrated by gradient ethanol and washed by distilled water. Some sections added DNase I at a dose of 1 μg/ml were regarded as positive control sample, and those treated without TdT were the negative ones. Under a 400-fold immunofluorescent microscope (wavelength 488 nm), the apoptotic cells were appeared yellow-green fluorescence in nucleus, and averaged in four random views in cortex and striatum, respectively.

### Immunohistochemical staining

Rabbit anti-rat TLR4, NFκB and TNFα moloclonal antibody (1:100) were purchased from Santa Cruz Co. Ltd. Strept-avidin-biotin complex (SABC) immunohisto-chemistry kit, diaminobenzidine (DAB) dye were granted by Bostor biological company in Wuhan China. Paraffin-embedded sections were deparaffinaged in dimethylbenzene, hydrated successively in gradient ethanol, restored antigen twice in a microwave oven. Immunohistochemical procedures were performed strictly according to the guidance of manufacture. Under a microscope, those with brown granules in cytoplasm or nucleus were considered as positive cells. And the slides added 0.1 mmol/L PBS (containing 1:200 non-immunity animal serum) instead of primary antibody had no response. Four serial sections were chosen from each experimental rat and observed randomly four views at cortex and striatum under a 400-fold microscope. Absorbance values (*A*) of each view was detected by a LEICA Qwin microgramme analytical system (Leica company).

### Enzyme linked immunosorbent assay (ELISA)

Rat TLR4 (No. E02T0013), NFκB p65 (No. E02N0014) and TNFα (No. E02T0012) ELISA kits were purchased from Blue Gene Co. Ltd. Five rats in each group were deeply anesthetized and decapitated at given time after MCAO/R. The ischemic hemisphere tissue (0.5 g) was quickly removed and ground fully into brain tissue homogenate. Then added normal sodium 500 μl, mixed well and centrifugalized for 10 minutes at 12000 r/min. The upper limpid liquid was collected and stored at -20°C (Avoid repeated freeze-thaw cycles). Prepare all standards before starting assay procedure. First, secure the desired number of coated wells in the holder, then add 50 μl of standards or samples to the appropriate well of the antibody pre-coated microtiter plate. Add 100 μl of conjugate to each well. Mix well, cover and incubate for 1 h at 37°C. Wash the microtiter plate 5 times using distilled or de-ionized water. Add 50 μl substrate A & B to each well. Cover and incubate for 15 minutes at 25°C. Add 50 μl of stop solution to each well. Mix well and calculate the mean absorbance value *A*_450 _for each set of reference standards and samples. The standard density is a X, the B/B0 is a Y, sitting to mark the paper in the logit-log up draw a standard curve. According to the B/B0 that need to be measured the sample can from sit to mark the density value that the paper looks up the sample up. The sensitivity by this assay is 1.0 pg/ml (TLR4 and TNFα) and 1.0 ng/ml (NFκB).

### Statistical Analysis

SPSS11.5 software was used for statistical analysis. Data were expressed as mean ± standard error ( ± s). Multi-group comparison was made by analysis of variance (ANOVA) and Student's test, and two-group comparison by *t*-test. Values were considered to be significant when *P *is less than 0.05.

## Results

### Neurobehavioral dysfunction score

There was no neurobehavioral dysfunction symptom in rats of the SO group, whose Bederson's score got 0 points. After cerebral ischemia/reperfusion injury, all animals showed neurobehavioral dysfunction. The Bederson's scores both in the SA group and PT group were obviously lower than that in the IC group (*F *= 24.90, *q *= 8.38-8.88, *P *< 0.01). The scores in PT group is slightly lower than that in SA group, but no significant difference was found between these two groups (*t *= 0.50, *P *> 0.05). Shown as table 1.

**Table 1:** Bederson's score, infarction volume and apoptotic cells ( ± s)

Groups	Bederson's score(n = 15)	Infarction volume(n = 5)	Apoptotic cells (n = 5)
SO group	0.00 ± 0.00	0.00 ± 0.00	3.53 ± 1.13
IC group	2.17 ± 0.35^Δ^	77.32 ± 3.06^Δ^	16.62 ± 3.25^Δ^
SA group	1.33 ± 0.43*	70.11 ± 3.13*	8.13 ± 2.15*
PT group	1.28 ± 0.38*^#^	68.73 ± 3.46*^#^	7.64 ± 2.08*^#^

^Δ^*P *< 0.01, vs the SO group; * *P *< 0.01, vs the IC group; ^# ^*P *> 0.05, vs the SA group.

### The cerebral infarction volume

**Figure 2:**
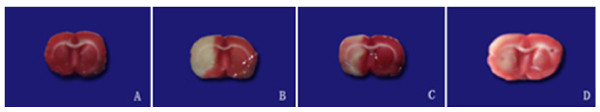
**The cerebral infarction volume shown by TTC stain (A. SO group, B. IC group, C. SA group, D. PT group)**.

By TTC stain, no cerebral ischemia infarction was shown in the brain slices of the SO group, while infarction lesion almost appeared in all the experimental rats after cerebral ischemic reperfusion injury. The volume of cerebral infarction in the SA group and PT group were significantly lower than that in the IC group (*F *= 30.76, *q *= 8.66-10.33, *P *< 0.01), but there is no statistical difference between the SA group and PT group (*t *= 1.66, *P *> 0.05). Shown as table 1 and figure 2.

### Neuronal apoptosis

**Figure 3:**
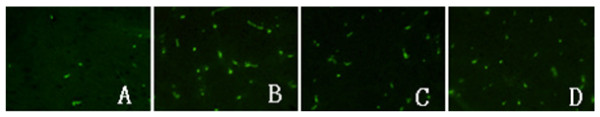
**Apoptosis in cortex shown by TUNEL × 200 (A. SO group, B. IC group, C. SA group, D. PT group)**.

A few apoptotic cells were scattered in the cortex and the striatum in SO group rats. Apoptotic cells were significantly increased in the IC group. As we expected, the number of apoptosis both in the SA group and PT group were obviously low compared with the IC group (*F *= 29.05, *q *= 4.03-12.84, *P *< 0.01), but no difference existed between the SA group and PT group (*t *= 0.49, *P *> 0.05). Shown as table 1 and figure 3.

### The expressions of TLR4, NFκB and TNFα

With the help of immunohistochemistry, we found that there were no region differences between the cortex and the striatum. Thus we calculated the absorbance values (*A*) in four confirmed views instead of the random ones in each brain slice.

**Figure 4:**
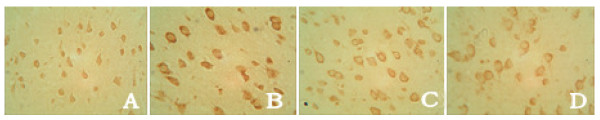
**TLR4 expression in cortex shown by SABC × 400 (A. SO group, B. IC group, C. SA group, D. PT group)**.

Few TLR4 positive cells with light yellow granules could be seen in the cortex and the striatum in SO group rats, and its expression was very weak. TLR4 expression in IC group was significantly elevated along with the increased *A *value as compared with the SO group rats; After the drug administration, the *A *values in the SA group and PT group were obviously decreased in contrast to the IC group rats (*F *= 4.33, *q *= 2.95-4.92, *P *< 0.05). Although the *A *value in the SA group was slightly lower than that in the PT group, there was no significant differences between the two treatment groups, suggesting that picroside 2 could inhibit the expression of TLR4 protein and play neuroprotective effects as same as salvianic acid A sodium (*t *= 1.31, *P *> 0.05). Shown as table 2 and figure 4.

**Table 2:** Absorbance values of TLR4, NFκB and TNFα expressions ( ± s)

Groups	n	TLR4	NFκB	TNFα
SO group	5	0.16 ± 0.09	0.14 ± 0.07	0.14 ± 0.08
IC group	5	0.46 ± 0.18^Δ^	0.65 ± 0.21^Δ^	0.51 ± 0.17^Δ^
SA group	5	0.28 ± 0.13*	0.26 ± 0.15*	0.30 ± 0.16*
PT group	5	0.24 ± 0.13*^#^	0.22 ± 0.08*^#^	0.25 ± 0.12*^#^

^Δ^*P *< 0.01, vs the SO group; * *P *< 0.01, vs the IC group; ^# ^*P *> 0.05, vs the SA group.

**Figure 5:**
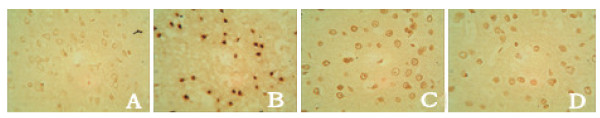
**NFκB expression in cortex shown by SABC × 400 (A. SO group, B. IC group, C. SA group, D. PT group)**.

Weak NFκB expression were shown in the cortex and the striatum in SO group rats. The number of NFκB positive cells rapidly increased, and its *A *value was significantly higher than that in the SO group; the expression of NFκB both in the SA group and PT group were apparently lower than that in the IC group (*F *= 14.25, *q *= 6.24-8.17, *P *< 0.01), but there was no difference between the SA group and PT group as shown in table 2 and figure 5 (*t *= 0.64, *P *> 0.05).

**Figure 6:**
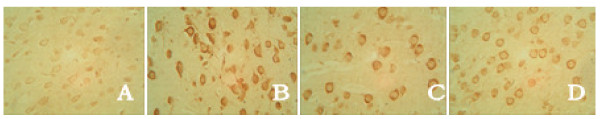
**TNFα expression in cortex shown by SABC × 400 (A. SO group, B. IC group, C. SA group, D. PT group)**.

Ditto, the TNFα expression was very weak in the cortex and the striatum in SO group rats. TNFα positive cells was clearly increased in the IC group, and its *A *value was significantly higher than that in the SO group; the *A *values in the SA and PT groups were obviously low compared with the IC group (*F *= 6.39, *q *= 3.42-6.03, *P *< 0.05), and there was no difference between the SA group and the PT group, too (*t *= 0.81, *P *> 0.05). Shown as table 2 and figure 6.

### The concentrations in brain tissue of TLR4, NFκB and TNFα

The concentrations in brain tissue of TLR4, NFκB and TNFα were lowly in SO group rats, and increased significantly in the IC group rats. In the SA group and PT group, the concentrations of TLR4 (*F *= 79.42, *q *= 7.20-21.43, *P *< 0.01), NFκB (*F *= 111.25, *q *= 7.98-25.27, *P *< 0.01) and TNFα (*F *= 95.29, *q *= 6.89-23.27, *P *< 0.01) were obviously lower than those in the IC group, but there was no difference between the SA group and PT group (*t *= 170, 1.01, 1.69, *P *> 0.05). Shown as table 3.

**Table 3:** The concentrations in brain tissue of TLR4, NFκB and TNFα ( ± s)

Groups	n	TLR4	NFκB	TNFα
SO group	5	14.26 ± 2.08	11.53 ± 2.13	12.34 ± 2.25
IC group	5	62.45. ± 7.26^Δ^	75.16 ± 9.12^Δ^	67.78 ± 8.37^Δ^
SA group	5	34.28 ± 5.04*	39.23 ± 4.38*	35.16 ± 4.71*
PT group	5	30.45 ± 4.32*^#^	31.64 ± 4.46*^#^	28.76 ± 4.03*^#^

^Δ^*P *< 0.01, vs the SO group; * *P *< 0.01, vs the IC group; ^# ^*P *> 0.05, vs the SA group.

## Discussion

TLR4 is a member of signal transduction family, which combines with the adapter protein MyD88 and then with interleukin-associated kinase (IRAKs) to cause NFκB translocation, to enable the innate immune and inflammatory responses related gene transcription, to activate the TLR4-MyD88 dependent signal transduction pathway and to induce inflammatory response[1]. Caso *et al *[2] found that the cerebral infarction volume in TLR4 deficient rats was smaller than that in normal TLR4 animals after middle cerebral artery occlusion and reperfusion. Hua *et al *[25] proved that cerebral infarction size in TLR4 knockout mice was also smaller than the wild-type ones, and its neurological deficit score was lower accordingly. The studies of Yang *et al *[26] showed that the TLR4 positive lymphomonocytes in blood circulation significantly elevated in patients with acute cerebral infarction as compared with that in the control group and the patients with transient ischemic attack, and the expression levels of TLR4 mRNA and TNFα in serum were consistent with IL6. From the data of correlation analysis, they further found that TLR4 and serum cytokine expression levels were closely related to the severity of stroke. Thus they speculated it is TLR4 that activates the downstream signaling protein, evokes the gene transcription associated with the encoding and inflammation-related factor, induces and aggravates the inflammatory response.

NFκB is an important nuclear transcription factor in eukaryotic cells, which exists in almost all the cells. It is located downstream of TLR signaling pathway, regulated by TLRs and involved in immune response and the processes of cell proliferation and differentiation. Cerebral ischemic reperfusion injury not only promotes the innate immune response of the immune system, but also activates TLR4-mediated signal transduction pathway to rapidly translocate NFκB from cytoplasm into nucleus, and combines with specific DNA sequence to stimulate downstream-associated factor (TNFα)-induced inflammation response and ischemia neuronal apoptosis[27]. With the application of MCAO models, Lou *et al *[28] confirmed that the increased TNFα protein and its mRNA expression and NFκB DNA-binding-activity could reduce the survival number of spinal cells in the hippocampal CA1 area, suggesting that NFκB activation can lead to neuronal apoptosis in inflammation following cerebral ischemic reperfusion injury. It will become a new treatment target to reduce cerebral ischemic reperfusion injury through efficiently decreasing TNFα protein and mRNA expression and inhibiting NFκB DNA binding-activity. Liu *et al *[29] reperted that the expression of NFκB enhanced in cytoplasmic or nuclear in brain slices, and the use of Oxymatrine decreased the NFκB expression and the infarction lesions in rats with permanent middle cerebral artery occlusion compared with the sham operation group. Webster *et al *[30] found that hypothermy could attenuate the NFκB inhibition factor (IκB) phosphorylation and IκB suppressor kinase, and decrease the expression of NFκB, thereby protect neurons in penumbra against focal ischemic reperfusion damage. The data of Zhang *et al *[31] have demonstrated that the intracerebroventricular administration of prostaglandin after cerebral ischemia could inhibit the expression of subunit NFκB and play the protection role in ischemia neurons. These results intimate that the inhibition of NFκB expression to reduce apoptosis may be one of the mechanisms to improve the brain ischemic reperfusion injury. Our previous researches proved that picroside 2 could inhibit the expressions of iNOS [16], NFκB and IκB[17], and reduce the expressions of Caspase-3, PARP[18] and to inhibit the neuronal apoptosis and thus improve the neurological function of rats with cerebral ischemia reperfusion injury[19]. It also reduced brain tissue edema and the aquaporin-4 (AQP4) expression, and the best therapeutic time window was 1 h after cerebral ischemic reperfusion [32].

This experiment showed that slight expressions of TLR4, NFκB and TNFα could be seen in the cortex and the striatum in the SO group, and their concentrations in brain tissue were very lowly. After cerebral ischemia 2 h and reperfusion 22 h, the expressions of these proteins, as well as the TUNEL-positive cells and concentrations in brain tissue increased significantly in contrast to the SO group. The data imply that the activation of TLR4 and its downstream NFκB inflammatory factor induce neuronal apoptosis and cerebral ischemic reperfusion injury. In this experiment, NFκB protein inactively expressed in cytoplasm in the SO group. After cerebral ischemic reperfusion, it mainly located in nuclear, suggesting that the activation of NFκB into nucleus promote the expression of the downstream target genes TNFα, be involved in inflammatory response and induce neuronal apoptosis. Accordingly, the experimental animals appeared cerebral infarction lesions, abnormal morphology and the increased neuronal apoptosis in ischemic ipsilateral, and neurobehavioral dysfunction in contralateral hemisphere. We also found that the injection of picroside 2 and salvianic acid A sodium via tail vein significantly reduced the amount of apoptotic cells, the concentrations in brain tissue and the expressions of TLR4, NFκB and TNFα proteins in the cortex and the striatum in MCAO/R rats, together with the decreased infarction size and the improvement of cellular structure and neurobehavioral function of rats. In short, the neuroprotective effect of picroside 2 and salvianic acid A sodium against cerebral ischemic reperfusion injury might be performed by inhibiting TLR4-NFκB signal transduction pathway to reduce inflammatory response-induced apoptosis. However, the concrete differences between the two Chinese drugs needs to further elucidated.

## Conclusion

Picroside 2 could down-regulate the expressions of TLR4, NFκB and TNFα to inhibit apoptosis and inflammation induced by cerebral ischemic reperfusion injury and improve the neurobehavioral function of rats.

## List of abbreviations used

MCAO/R: middle cerebral artery occlusion/reperfusion; SO group: sham operated group; IC group: ischemia control group; SA group: salvianic acid A sodium group; PT group: picroside treated group; TTC: tetrazolium chloride; TLR4: toll-like receptor 4; NFκB: nuclear transcription factor κB; TNFα: tumor necrosis factor α; TUNEL: terminal deoxynucleotidyl transferase-mediated biotinylated deoxyuridine triphosphate nick end labelling; SABC: strept-avidin-biotin complex; DAB: diaminobenzidine; ELISA: enzyme linked immunosorbent assay.

## Competing interests

The authors declare that they have no competing interests.

## Authors' contributions

GYL was responsible for design, experiment and manuscript writing. XXY, LQ, LZ and DF carried out the experiments, data acquisition and analysis. All authors read and approved the final manuscript.
